# Utility of Intranasal Tapentadol in Redefining Postoperative Pain Management in Total Knee Arthroplasty: A Prospective Observational Study

**DOI:** 10.7759/cureus.73220

**Published:** 2024-11-07

**Authors:** Prashant Kamble, Sameer Panchal, Rudra M Prabhu, Shubhranshu Shekhar Mohanty, Rohan Dhotre

**Affiliations:** 1 Orthopaedics, Seth Gordhandas Sunderdas (GS) Medical College and King Edward Memorial (KEM) Hospital, Mumbai, IND; 2 Orthopaedics, Sir HN Reliance Foundation Hospital and Research Centre, Mumbai, IND

**Keywords:** intranasal, opioids, postoperative pain management, tapentadol, total knee arthroplasty

## Abstract

Introduction

Effective strategies to minimize postoperative pain following total knee arthroplasty (TKA) are essential to improve functional outcomes. This study aimed to evaluate the effectiveness and safety of tapentadol nasal spray as a form of patient-controlled analgesia (PCA) for postoperative pain management after TKA. The intranasal route was chosen for the study as intranasal tapentadol has been shown to have superior pain reduction as compared to intravenous tapentadol. Intranasal instillation of tapentadol is rapid and more effective than the parenteral or oral route. Additional advantages of the intranasal route include enhanced comfort, convenience, and safety.

Methods

The present study was a single-center prospective observational study including 120 patients undergoing unilateral TKA who were administered tapentadol nasal spray post-surgery (22.5 mg of tapentadol per spray). Pain was objectively assessed using the visual analog scale (VAS) on postoperative days (POD) 1, 2, and 3, before and after spray administration. The pain severity was graded into mild (VAS 1-3), moderate (VAS 4-6), and severe (VAS 7-10) based on the VAS score. The time duration required for the pain severity to become mild from the pre-spray level post-administration of the nasal spray was recorded on all three PODs. The time required in hours for the pain severity to worsen from mild (VAS 1-3) to moderate (VAS 4-6) or severe (VAS 7-10) was also recorded on all three PODs. The statistical analysis plan for this study involved the analysis of VAS scores collected on PODs 1, 2, and 3. Categorical variables were expressed as percentages, while numerical variables were presented as means and standard deviations. The significance of differences between pre and post-treatment VAS scores was analyzed using Student’s t-test. Differences between proportions were analyzed using the Chi-square or Fisher's exact test. The Kolmogorov-Smirnov test was used to test the normality of the quantitative data. The Analysis of Variance (ANOVA) test was applied to compare the means across the three PODs. A two-tailed significance level of 0.05 was set for all tests to determine statistical significance.

Results

The mean pre-spray VAS scores recorded on POD 1, 2, and 3 were 8.07, 7.64, and 7.40 respectively. The mean post-spray VAS scores recorded on POD 1, 2, and 3 were 4.63, 4.71, and 3.95 respectively. There was a statistically significant reduction in the VAS scores on each of the three days when measured before and after spray administration (p<0.001). The average time needed for the pain severity to become mild from the pre-spray level in minutes on POD 1, 2, and 3 was 14.07, 13.36, and 12.34 respectively. Thus, this metric significantly declined (p<0.001) from POD 1 to POD 3. The time taken in hours for the pain severity to worsen from mild to moderate or severe on POD 1, 2, and 3 was 6.57, 6.70, and 6.98 respectively indicating that there was a significant increase in the time till the pain severity worsened from POD 1 to POD 3 (p<0.001)*. *There were no major drug-induced adverse reactions following the administration of intranasal tapentadol.

Conclusion

Intranasal tapentadol spray (22.5 mg per spray) is an acceptable modality of postoperative pain management in patients undergoing TKA. It has a long-lasting effect, rapid onset, minimal side effects, and can be self-administered by the patient.

## Introduction

Postoperative pain management after total knee arthroplasty (TKA) remains a formidable challenge, central not only to patient comfort but also to facilitating early mobilization, reducing postoperative complications, and promoting expedited return to normal activities. Traditionally, systemic opioids have been the cornerstone of postoperative pain management in TKA, offering effective pain relief but associated with significant side effects. These limitations necessitate the exploration of alternative analgesic strategies that afford effective pain relief with fewer side effects. Tapentadol, with its unique dual mechanism of action combining μ-opioid receptor agonism and norepinephrine reuptake inhibition, provides effective pain control with a reduced risk of opioid-related side effects. There has been limited research regarding the delivery of tapentadol via the intranasal route. It has been shown previously that intranasal tapentadol is an acceptable choice for the management of postoperative pain as it provides improved pain reduction and favorable sleep outcomes [1]. Moreover, when compared to intravenous paracetamol, intranasal tapentadol provides a greater reduction of postoperative pain in patients undergoing lower limb orthopedic surgeries [2]. Therefore, the study aimed to analyze the efficacy, tolerability, and adverse effects of intranasal tapentadol in patients undergoing elective unilateral TKA and determine its role as a form of patient-controlled analgesia (PCA).

## Materials and methods

Approval for the study was obtained from the Institutional Review Board of Seth GS Medical College and KEM Hospital (IRB approval number CT/TAPE/PAIN/20/04_01). This prospective observational study was conducted at Seth GS Medical College and KEM Hospital, a tertiary care center, from April 2021 to April 2022. A well-written informed consent was obtained from all participants, guaranteeing awareness of the study's nature, potential risks, and rights, including withdrawal without affecting their care. The procedures followed were as per the Helsinki Declaration of 1975. The methodology incorporated patient selection based on defined criteria, a standardized tapentadol administration protocol, and rigorous pain assessment methods. Our study was structured as a single-center, prospective observational study, and the study design ensured consistent patient care and data collection. The study drug was prescribed to the patients who had undergone TKA.

The primary goal was to measure tapentadol's effectiveness in reducing postoperative pain via Visual Analog Scale (VAS) scores immediately after surgery, with secondary objectives assessing pain relief duration and any adverse effects. We included a total of 120 patients undergoing elective unilateral TKA, of which 92 patients had primary degenerative osteoarthritis, 14 patients had post-traumatic arthritis, eight patients had rheumatoid arthritis, and the remaining six patients had post-infective arthritis. The patients were selected as per pre-defined inclusion and exclusion criteria (Table 1).

**Table 1:** Inclusion and exclusion criteria for the study

Inclusion criteria	Exclusion criteria
Age 50-75 years	Patients <50 or >75 years of age
Patients undergoing unilateral total knee replacement	Patients undergoing unicompartmental knee replacement, bilateral total knee replacement, and revision total knee replacement
Patients who gave consent for spinal anesthesia	Patients who refused to give consent for spinal anesthesia or in whom spinal anesthesia was contraindicated
	Patients in whom intranasal tapentadol spray was contraindicated (severe asthma or breathing problems, bowel obstruction, history of consumption of Monoamine oxidase inhibitor drugs in the past two weeks, history of hypersensitivity to tapentadol or any of the excipients)

A total of 14 patients required additional analgesia over and above intravenous paracetamol and intranasal tapentadol and were excluded from the study. Expecting a minimum difference of 20% reduction of VAS score after the use of tapentadol spray, with a 95% confidence interval and 80% power, the minimum sample size deduced was 95. Considering attrition of 20%, we increased our sample size to 114. However, for our convenience, we included 120 cases in the present study.

All the surgeries were performed under plain spinal anaesthesia by a single experienced arthroplasty surgeon (SM) with a standard medial parapatellar approach utilizing a tourniquet. All the patients were given standard postoperative nausea and vomiting prophylaxis in the form of a single intravenous dose of dexamethasone 8 mg after induction of anaesthesia and ondansetron postoperatively on a PRN basis**.** A standardized intraoperative periarticular cocktail infiltration (150 ml) prepared using a combination of bupivacaine (0.25%, 40 mL), fentanyl (100 μg, 2 mL), clonidine (150 μg, 1 mL), cefuroxime (750 mg, 10 mL), and normal saline (0.9%, 97 mL) was given to all patients before final closure. All patients received a post-operative intravenous infusion of paracetamol with a dose calculated as per the weight (15 mg/kg QID, with a maximum permissible dose of 4 grams). No other analgesic drugs in any form were used apart from tapentadol nasal spray. None of the participants received a pre or post-operative analgesic block. All the patients were admitted to the ward for a minimum of three days post-surgery. The spray used in the present study was TAPEASE NS, marketed by Torrent Pharmaceuticals Ltd. The contents of the spray were tapentadol hydrochloride 225 mg/ml and benzalkonium chloride solution (50%) as a preservative. Each spray (0.1 ml) contained 22.5 mg of tapentadol hydrochloride. Dosing specifics aligned with manufacturer guidelines and best practice pain management protocols. Once the patient arrived to the ward, the pre-spray VAS score was calculated. Eligible patients were given a single intranasal dose of tapentadol (one spray in each nostril, a total of 45 mg) starting one hour after surgery, and post-spray VAS scores were recorded 15 minutes after administration of the spray. As per Chang et al., immediate-release (IR) oral tapentadol has been shown to have a quick analgesic effect at 30 minutes [3]. We recorded the scores 15 minutes after administration of the spray, in line with our hypothesis that intranasal tapentadol has a faster onset of action. The superior pharmacodynamic profile of intranasal tapentadol was also shown by Javia and Thakkar in their study[4]**.** The severity of pain was graded as per the VAS score as follows: 0 = no pain, 1-3 = mild, 4-6 = moderate, and 7-10 = severe pain [5]. The time duration required for the pain severity to become mild from the pre-spray level was recorded on all three PODs. The time required in hours for the pain severity to worsen from mild (VAS 1-3) to moderate (VAS 4-6) or severe (VAS 7-10) was also noted on three consecutive PODs.

The statistical analysis plan for this study involved the analysis of VAS scores collected on PODs 1, 2, and 3. Data were analyzed using the software EPI Info version 7.2. Categorical variables were expressed as percentages, while numerical variables were presented as means and standard deviations. The significance of differences between pre and post-treatment VAS scores was analyzed using Student’s t-test, and differences between proportions were analyzed using the Chi-square or Fisher's exact test. The Kolmogorov-Smirnov test was used to test the normality of the quantitative data, while the Analysis of Variance (ANOVA) test was applied to compare the means across the three PODs. Repeated measures ANOVA was applied to test the difference in the means of VAS scores over three consecutive PODs (POD 1, POD 2, and POD 3) before and after the administration of tapentadol nasal spray. Specifically, repeated measures ANOVA was utilized to test whether there were significant changes in the mean VAS scores on each POD. The results demonstrated a statistically significant reduction in pain levels, as evidenced by p-values below 0.001 across all three days. This statistical test was appropriate for the study design, as it involved repeated measurements of pain levels within the same group of patients over time.A two-tailed significance level of 0.05 was set for all tests to determine statistical significance.

## Results

A total of 120 patients were included in the study with a mean age of 65.84 years (ranging from 50 to 75 years) (Table 2).

**Table 2:** Sample size distribution based on age groups SD: Standard deviation

Age group (years)	Frequency	Percentage
50-60	22	18.33
61-70	60	50.00
>70	38	31.67
Total	120	100.00
Mean	65.84	
SD	5.83	

The study included 77 females (64.16%) and 43 males (35.83%) (Table 3).

**Table 3:** Distribution of the population in terms of gender

Gender	Frequency	Percentage
Female	77	64.16
Male	43	35.83
Total	120	100.00

**Figure 1:**
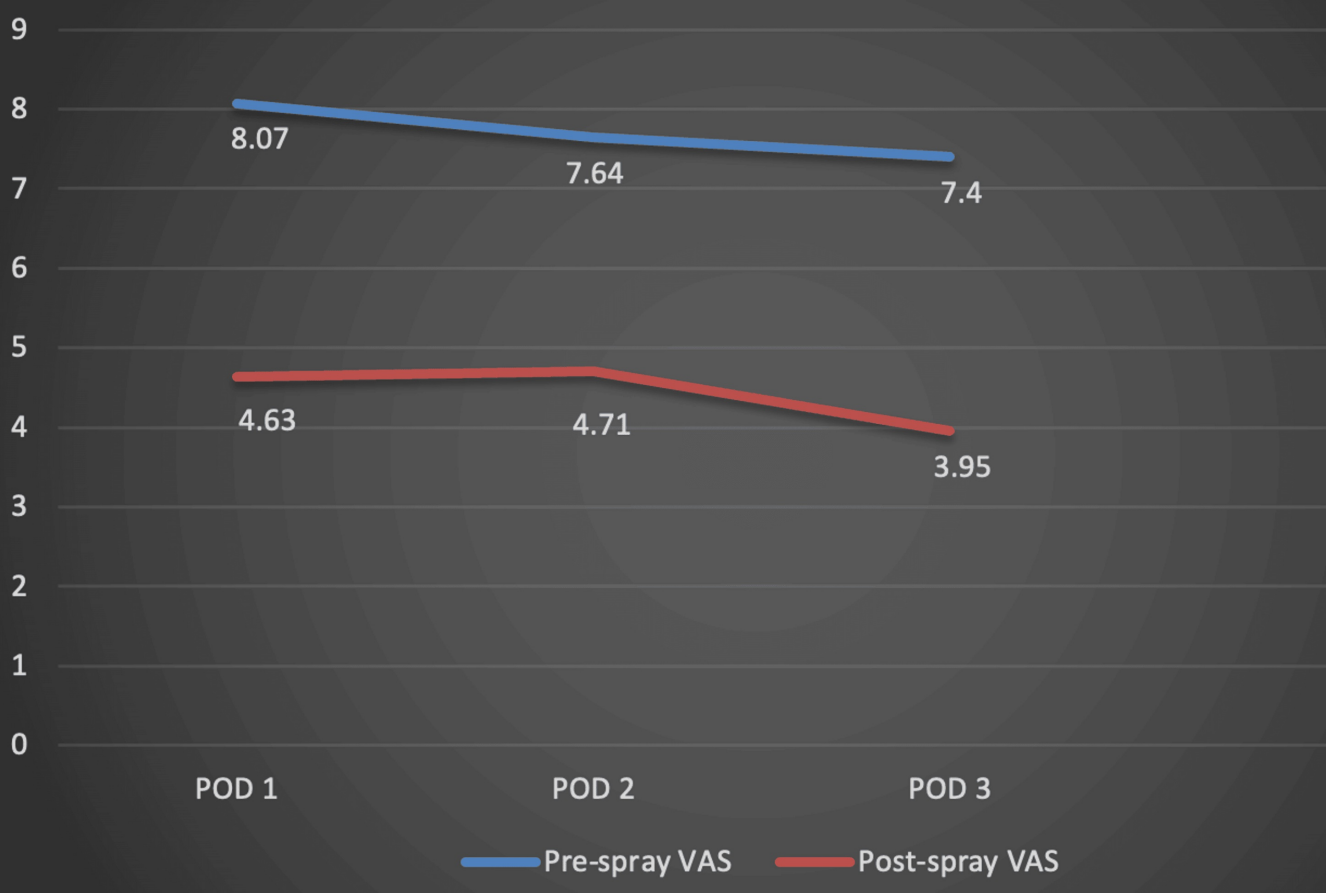
Graphical representation of the variation in pre- and post-spray VAS scores over the three postoperative days. POD: Postoperative Day; VAS: Visual Analogue Scale

Out of 120 patients, 68 patients underwent left TKA (56.67%) and 52 patients underwent right TKA (43.33%). Analysis of the pre-spray and post-spray VAS scores recorded on POD 1, 2, and 3 revealed that there was a statistically significant reduction in the VAS scores on each of the three days when measured before and after administration of the spray (p<0.001) (Table 4, Figure 1).

**Table 4:** Comparison of mean VAS scores before and after administration of tapentadol spray on postoperative days 1, 2, and 3. *A p-value of <0.05 was considered significant. The pre-spray and post-spray VAS values have been represented as Mean±SD. The significance of differences between pre- and post-treatment VAS scores was analyzed using Student’s t-test. SD: Standard Deviation; VAS: Visual Analogue Scale

Postoperative Day	Pre-spray VAS Mean (SD)	Post-spray VAS Mean (SD)	Percentage Reduction	p-Value
Day 1	8.07 (0.80)	4.63 (1.53)	42.63%	<0.001*
Day 2	7.64 (0.80)	4.71 (1.02)	38.35%	<0.001*
Day 3	7.40 (0.83)	3.95 (1.00)	46.62%	<0.001*

**Figure 2:**
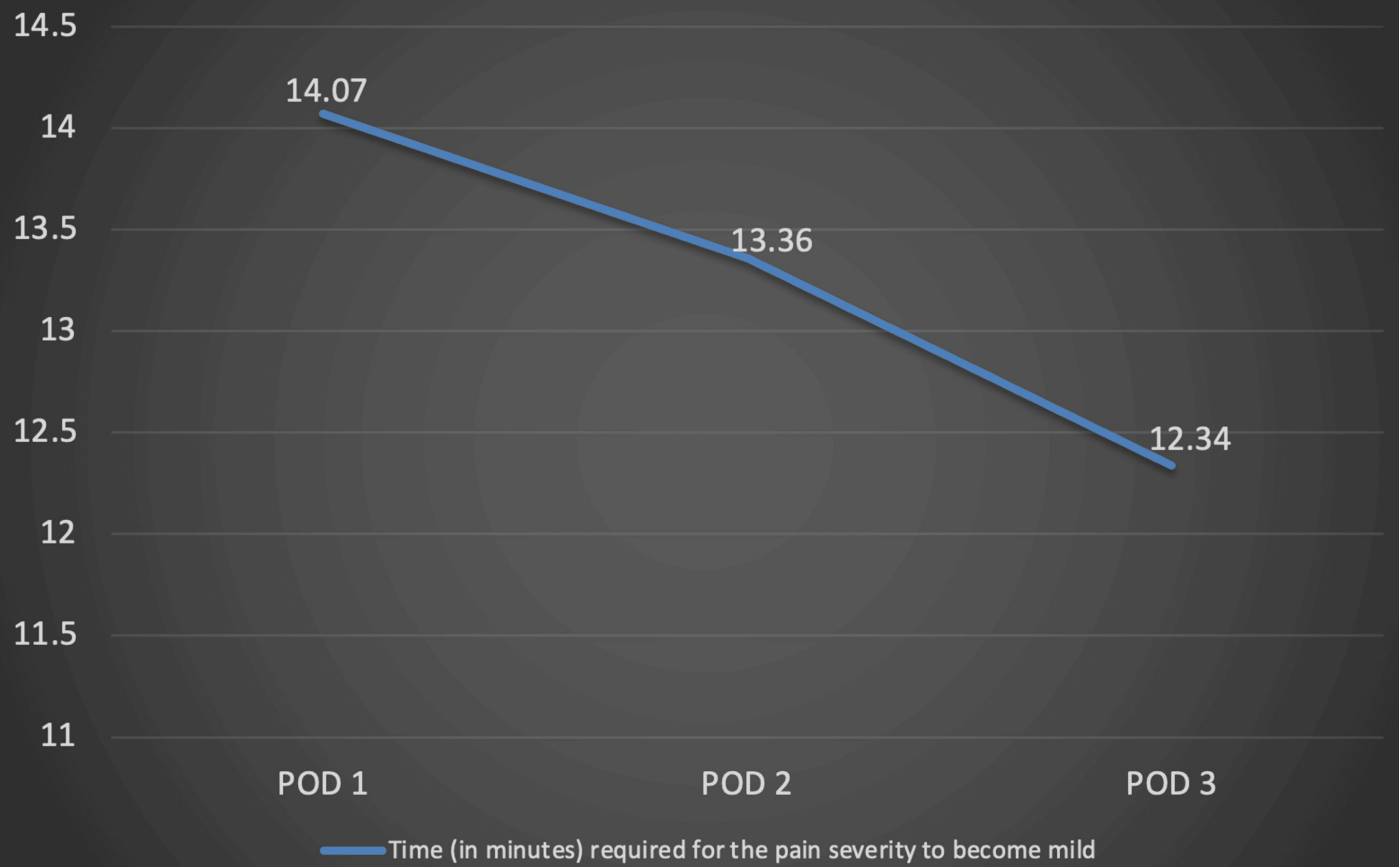
Graphical representation showing the decline in time required to achieve for the pain severity to become mild with each successive postoperative day POD: Postoperative day

**Figure 3:**
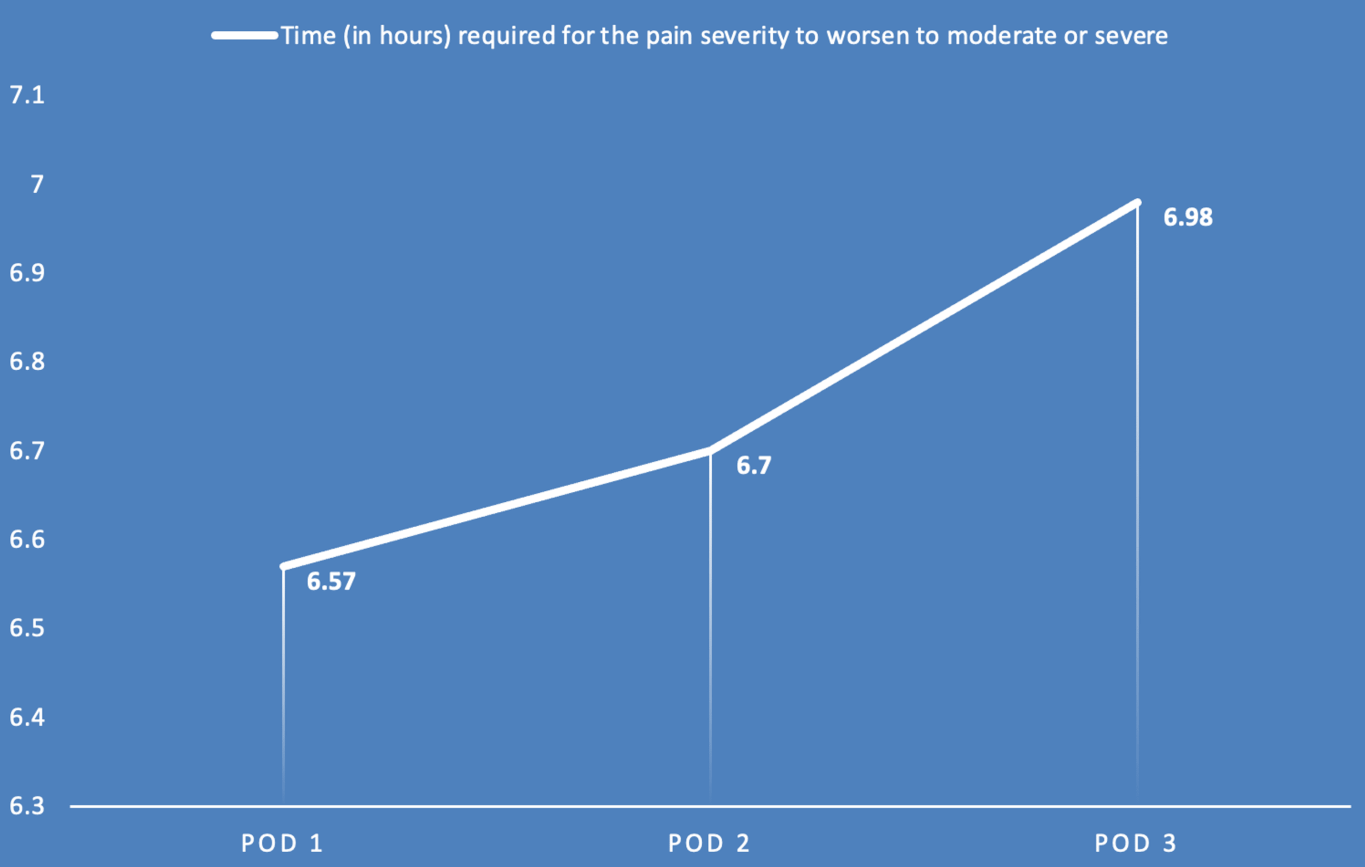
Graphical representation showing the increasing duration for the pain severity to worsen on successive postoperative days. POD: Postoperative Day

Further analysis revealed that there was a statistically significant decline (p<0.001) in the time required in minutes for the pain severity to become mild (VAS 1-3) from the pre-spray level after administration of intranasal tapentadol from POD 1 to POD 3. There was also a statistically significant increase in the time required for the pain severity to worsen from mild (VAS 1-3) to moderate (VAS 4-6) or severe (VAS 7-10) (p<0.001) (Table 5, Figures 2, 3).

**Table 5:** Tabular representation of the time required for the pain severity to become mild from the pre-spray level after administration of tapentadol (Row 1) and the time duration required for the pain severity to worsen from mild to moderate or severe (Row 2) on postoperative days 1, 2, and 3 respectively. *A p-value of <0.05 was considered significant. The time required for the pain severity to become mild from the pre-spray level and the time required for the pain severity to worsen from mild to moderate or severe have been represented as Mean±SD. The ANOVA (Analysis of Variance) test was applied to compare the means across the three PODs. VAS: Visual Analogue Scale

Parameter	Day 1; Mean (SD)	Day 2; Mean (SD)	Day 3; Mean (SD)	p-value
Time (in minutes) required for the pain severity to become mild (VAS 1-3) from the pre-spray level	14.07 (3.09)	13.36 (2.78)	12.34 (2.42)	<0.001*
Time (in hours) required for the pain severity to worsen from mild (VAS 1-3) to moderate (VAS 4-6) or severe (VAS 7-10)	6.57 (1.56)	6.70 (1.07)	6.98 (0.98)	<0.001*

All the patients were subjected to intra-nasal tapentadol spray for the first time. No serious adverse events and anaphylaxis were recorded. Twenty-four patients experienced some nasal irritation after the first instilment which resolved on its own after a few minutes. ​​Nausea was observed in 16 patients out of 120 as a self-reported issue on day 1. It settled down in all the patients by POD 3, with no further requirement of anti-emetics. None of the patients discontinued tapentadol or had a dose reduction due to serious adverse events, and none exhibited withdrawal symptoms. There were no episodes of somnolence or delirium which required treatment discontinuation or dose reduction.

## Discussion

The complexities of post-TKA pain management highlight the necessity for innovative solutions to overcome current obstacles. Acute postoperative pain in patients undergoing TKA delays early ambulation, thereby increasing the risk of thromboembolism and harming the overall outcome of the surgery [6]. The incidence of severe and moderative postoperative pain in these patients is 60% and 30% respectively [7], with many patients refusing surgical treatment because of the fear of postoperative pain [8]. The currently employed approaches for managing acute postoperative pain are preoperative regimens that include opioids and cyclooxygenase-2 inhibitors, intraoperative regimens such as local infiltration analgesia, and postoperative regimens such as nerve blocks, epidural analgesia, and PCA. PCA delivers continuous analgesia per the patient’s requirements, leading to better pain control and improved satisfaction rates [9-11]. It involves using a pre-programmed pump that enables the patient to administer analgesia independently, generally via the intravenous (IV) route. Although this alternative has been shown efficacious in mitigating acute postoperative pain after TKA, its disadvantages include vomiting, nausea, constipation, retention of urine, and respiratory depression [12]. An alternative to IV PCA is the administration of PCA via the subcutaneous route. However, it has been shown that although subcutaneous PCA is safe and effective for TKA patients, it does not promote early functional recovery [13]. Another alternative route for delivering PCA is the intranasal route. Tapentadol is a centrally-acting analgesic that can be delivered via the intranasal route and offers advantages such as rapid absorption, ease of use, and fewer systemic side effects compared to traditional methods, making it a compelling choice for pain management. The present study is an attempt to determine the efficacy of intranasal tapentadol as a modality of postoperative analgesia in patients undergoing TKA. We hypothesized that intranasal tapentadol spray would be an effective and safe form of PCA for patients undergoing TKA, with a rapid onset and long-lasting action to tackle early postoperative pain and improve functional outcomes.

Tapentadol is a unique analgesic having a two-fold action, combining the properties of mu-opioid receptor (MOR) agonism and norepinephrine reuptake inhibition (NERI). It has an improved side effect profile when compared to conventional opioids and nonsteroidal anti-inflammatory drugs, similar analgesic efficacy as compared to oxycodone, and better gastrointestinal tolerability than full mu-opioid receptor agonists. The efficacy, mode of action, adverse effect profile, and pharmacokinetic and pharmacodynamic properties of tapentadol have been extensively studied since its advent [14, 15]. Tapentadol’s additional mechanism of action (i.e., inhibition of norepinephrine reuptake via α2-adrenoceptors) enables it to alleviate both nociceptive pain and neuropathic pain [16, 17]. This unique characteristic distinguishes tapentadol from most other strong/WHO step III opioids (i.e., morphine and fentanyl) that are predominantly mu-opioid receptor agonists [18].

Tapentadol has been studied exhaustively as a form of postoperative analgesia. Three randomized, double-blinded, phase-three studies have critically evaluated the effectiveness of tapentadol in an oral molecular form for the relief of moderate to severe acute pain [19-21]. The trials have shown that the side-effect profile of tapentadol is quite favorable with exceptional patient tolerability and compliance. In another randomized, double-blinded, placebo-controlled study, Kleinert et al. compared the efficacy of oral doses of tapentadol (25, 50, 75, 100, or 200 mg), morphine sulfate (60 mg), ibuprofen (400 mg), and placebo in patients undergoing mandibular third molar extraction [21]. They deduced that a single oral dose of tapentadol (75 mg or higher) effectively abated moderate-to-severe postoperative dental pain in a dose-dependent manner with a favorable side-effect profile and was well-tolerated compared to morphine.

A systematic review and meta-analysis concluded that 75 mg of immediate-release tapentadol might be an optimal dose for moderate to severe pain control with fewer side effects [22]. Considering all the dosing formulations, tapentadol had comparable efficacy to oxycodone with much better tolerance and minimal incidence of total adverse events [22]. Contradictory results were seen in a randomized controlled trial as the addition of extended-release tapentadol molecule to a multi-modal analgesia regimen did not significantly improve pain reduction when compared to controlled-release oxycodone or placebo [23]. The only advantage in the tapentadol group was a better safety profile with a lesser prevalence of constipation. A study comprising three double-blind randomized trials concluded that immediate-release (IR) tapentadol has potent analgesic action and is well tolerated for moderate to severe postoperative pain. All three trials stated that IR tapentadol showed a statistically significant improvement in pain control compared with placebo and results were almost equivalent to common opioids including morphine or oxycodone IR [24]. A novel open-label, Phase 3b study concluded that prolonged-release oral tapentadol was highly effective and acceptable in patients with severe degenerative knees compared to WHO step III opioids [25].

An advantage of tapentadol is that it can be given effectively via the intranasal route. A novel in-vivo research paper on forming chitosan nanoparticles (CS-NPs) loaded with tapentadol hydrochloride and transporting the molecule from the nose to the brain analyzed the efficiency of the nasal delivery system over other channels. The authors concluded that intranasal tapentadol-loaded CS-NPs can effectively transport the molecule to the brain through the respiratory tract and may serve as a non-invasive means of drug delivery to the brain [4]. The study suggested that intranasal instillation of tapentadol-loaded CS-NPs delivers the drug rapidly and more effectively to the brain as compared to the parenteral or oral route.

Our study highlights the crucial role of effective postoperative pain relief provided by intranasal tapentadol, demonstrated by statistically significant reductions in VAS scores measured before and after administration of the spray on each of the three PODs. The mean pre-spray VAS scores recorded on POD 1, 2, and 3 were 8.07, 7.64, and 7.40 respectively, while the mean post-spray VAS scores recorded on POD 1, 2, and 3 were 4.63, 4.71, and 3.95 respectively (p<0.001). The average duration in minutes required for the pain severity to become mild (VAS 1-3) from the pre-spray level after tapentadol administration on POD 1, 2, and 3 was 14.07, 13.36, and 12.34 respectively (p<0.001). This statistically significant decline provided evidence of tapentadol's rapid onset of action. Further assessment of the average duration in hours till the pain severity worsened from mild (VAS 1-3) to moderate (VAS 4-6) or severe (VAS 7-10) showed that the duration was 6.57, 6.70, and 6.98 respectively on POD 1, 2, and 3 (p<0.001)**.** This metric was crucial for understanding the longevity of pain relief tapentadol provides, which is essential for patient comfort and satisfaction during the critical early recovery phase. Overall, the findings of our study showed that intranasal tapentadol is effective, has a rapid onset of action, long-lasting analgesic effect, and can be safely self-administered by the patient. This would make it an acceptable modality of PCA without the associated risk of dependence seen with long-term use of opioids. In the modern era of fast-track arthroplasty, intranasal tapentadol could be a game changer in postoperative pain management by facilitating early recovery post-TKA and thus, a shortened hospital stay.

The strength of our study was its prospective observational nature. While our findings are robust and indicate a clear benefit of intranasal tapentadol for post-TKA pain management, acknowledging the study's limitations paves the way for future research. The relatively smaller sample size and the lack of a control group were the limitations of our study. Subsequent studies should aim to compare intranasal tapentadol directly with other analgesics and administration methods, explore long-term outcomes beyond the immediate postoperative period, assess its role in reducing opioid dependence in a broader patient population, and also determine its cost-effectiveness.

## Conclusions

Intranasal tapentadol has a rapid onset of action and provides long-lasting analgesia after TKA*.* Lesser levels of pain post-TKA considerably aid the process of rehabilitation, which is the cornerstone of achieving favorable functional outcomes. Intranasal tapentadol offers a non-invasive, patient-centric method of pain management that aligns with the current healthcare emphasis on minimizing opioid exposure and enhancing patient autonomy. This study sets the stage for exploring the novel tapentadol molecule in post-operative pain management strategies after TKA, which could eventually lead to improved patient outcomes and a shift towards more judicious opioid use.
